# Peculiar conduction

**DOI:** 10.1007/s12471-025-01976-x

**Published:** 2025-08-27

**Authors:** Vladimir D. C. L’Espoir, Reinder Evertz, Rypko Beukema

**Affiliations:** Department of Cardiology, Radboud University Medical Centre, Nijmegen, The Netherlands

A 72-year-old man with a history paroxysmal atrial fibrillation presented for his annual cardiology follow-up visit. He reported feeling well, with no complaints or symptoms. Clinical examination revealed normal findings, and the resting electrocardiogram (ECG) is shown (Fig. 1). The patient was referred to the emergency department for telemetric observation and further analysis.

**Fig. 1:**
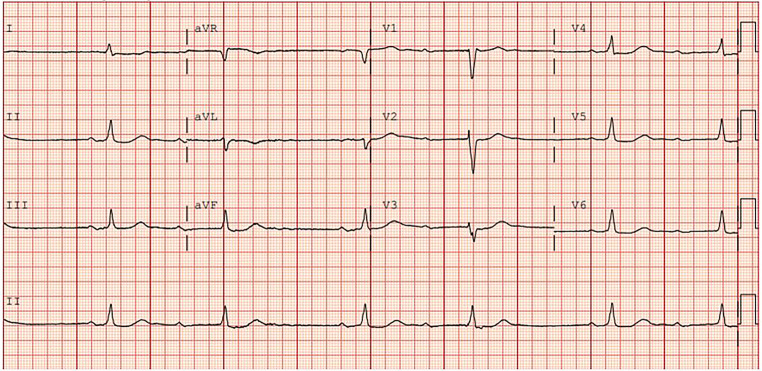
12-lead resting electrocardiogram performed at the outpatient clinic

What rhythm(s) are observed on this ECG? And how would you describe the AV conduction?

## Answer

You will find the answer elsewhere in this issue.

